# CRISPR mutant rapid identification in *B. napus*: RNA-Seq functional profiling and breeding technology application

**DOI:** 10.3389/fpls.2025.1572020

**Published:** 2025-04-22

**Authors:** Rui Geng, Xiang Fan, Rehman Sarwar, Yong Wang, Ke Dong, Xiao-Li Tan

**Affiliations:** ^1^ School of Food and Biological Engineering, Jiangsu University, Zhenjiang, China; ^2^ School of Life Sciences, Jiangsu University, Zhenjiang, China

**Keywords:** *Brassica napus* L., CRISPR/Cas9, IDA-HAE/HSL2 signaling pathway, rapid identification, RNA-Seq

## Abstract

**Introduction:**

Traditional rapeseed breeding is inefficient and imprecise. CRISPR genome editing offers a precise alternative for trait improvement. Here, we edited the *Bnaida* gene in elite rapeseed cultivar ZS11 to study its role in floral organ abcission and enable rapid trait transfer to elite lines.

**Methods:**

The *BnaIDA* gene was CRISPR-edited in ZS11. Phenotypes (petal adhesion time, cracking force of siliques) were statistically analyzed. And analyze the mutants using RNA -Seq. Edited alleles were introgressed into elite line SW1-6 via backcrossing. Locus-specific primers enabled efficient genotyping to distinguish hetero- and homozygous plants during selection.

**Results and discussion:**

In this study, The *Bnaida* mutant by gene editing in the cv ZS11, which is widely used in rapeseed breeding. The phenotypic analysis showed that the petal was attached to the pod and pods were harder to crack in edited plants, and then we quickly introduced two *Bnaida* loci into the elite line of SW1-6 by backcrossing with edited ZS11 as the donor plant. Locus-specific primer combinations were designed to differentiate heterozygous and homozygous genotypes in backcrossing generations, enabling efficient and rapid selection. This study highlights the integration of gene editing and genotyping selection, offering insights into the future of gene editing-assisted breeding.

## 1 Introduction

The cultivation and improvement of rapeseed, named *Brassica napus.* L, known as a crucial oilseed crop worldwide, are impeded by several breeding challenges including the enhancement of oil quality, the augmentation of stress tolerance and the optimization of yield. However, traditional breeding approaches for rapeseed have unavoidable weaknesses owing to its long operating period and low efficiency. *Brassica napus* is a widely grown oil crop that could be cultivated worldwide. In particular, *Brassica napus* (AACC, 2n=38), evolved from the hybridization of *Brassica rapa* (AA, 2n=20) and *Brassica oleracea* (CC, 2n=18), is an allotetraploid hybrid and belongs to the Cruciferae, along with *Arabidopsis* (Chalhoub et al., 2014). The complex genome of *Brassica napus*, characterized by numerous homologous genes, poses challenges in generating effective and specific genotype and phenotype mutations compared to Arabidopsis. This complexity necessitates the mutation of multiple genes to achieve distinct phenotypic changes. Recently, the CRISPR/Cas9 gene-editing system has been successfully applied in various crops, including *B. napus*, to address issues related to low yield and disease susceptibility, thereby offering significant solutions in modern agriculture. Previously, three genes (*SlER*, *SP5G* and *SP*) controlling stem length, flowering and fruiting time of tomato were modified by gene editing tools, creating a new type of “urban tomato” by integrating excellent traits (Kwon et al., 2020).Genome editing of the Glucan, Water-Dikinase 1(GWD1) in rice showed its good application potential to improve the yield and quality of rice at the same time (Wang et al., 2021). Gene editing is also widely used in *B.napus*, the knockout of *BnaLPAT2/5* could change the oil body size and fatty acid content, then gene editing of *BnaQCR8* could improve the resistance to two fungi and would not affect other good agronomic traits (Zhang et al., 2019, 2021). CRISPR/Cas9 technique offers an opportunity to knockout the gene precisely in one round selection, which showed advantage over traditional breeding method. Utilization of CRISPR/Cas9 system played an important role in recent researches in molecular mechanisms and trait improvement. For example, knocking out *BnaTT2* and *BnaTT8* boosts seed oil content by up to 5% and reduces lignin and pigment (Li et al., 2024). Editing *BnaC07.GLIP1* improves resistance to *Sclerotinia sclerotiorum* by changing phospholipid metabolism and ROS balance (Ding et al., 2024). CRISPR/Cas9 technique is suitable for editing polyploid genomes like B. napus, where it needs to knock out multiple homologs all in one step. For instance, editing *BPM6* and *DMR6* gives broad disease resistance with over 60% efficiency (Zhang et al., 2025). The precision in CRISPR/Cas9 meditated gene editing reduces off-target effects and ensures stable traits which is suitable for experiment and breeding process.

However, challenges remain in using Agrobacterium-mediated transformation (AF) for stable gene editing, especially with rapeseed, due to compatibility issues with tissue culture and biosafety concerns. Despite the prevailing reliance on Agrobacterium-mediated transformation for achieving stable gene editing in plants, certain challenges persist, particularly with rapeseed (Dhugga, 2022). A substantial number of rapeseed breeding materials exhibit compatibility issues with tissue culture techniques, followed by the associated biosafety concerns which could not be overlooked. In light of these challenges, an alternative breeding paradigm has emerged (Hao et al., 2020). First, gene editing is performed on transformable rapeseed varieties, followed by self-pollination to produce plants without exogenous T-DNA. Secondly, a series of hybridization and backcrossing steps is applied with other varieties.

Premature abscission of floral organs or silique is a major constraint on crop production, especially in *Brassica napus*, which causes the spread of *Sclerotinia sclerotiorum* and the loss of yields of up to 50% in mechanised harvesting (Li et al., 2021). Furthermore, the flowering of rapeseed is becoming more and more attractive to tourists, and the annual comprehensive tourism revenue of the Rapeseed Flower Tourism Festival could reach several billion RMB (Zhang et al., 2021). Therefore, the development of new germplasm incorporating multiple desirable traits is essential for the advancement of the rapeseed industry in contemporary agriculture.

In particular, IDA (INFLORESCENCE DEFICIENT IN ABSCISSION) and IDA-LIKE(IDL) proteins control floral organ abscission in *Arabidopsis* (Butenko et al., 2003; Santiago et al., 2016). In our previous study, we used the CRISPR/Cas9 system to precisely edit the two IDA homologous genes BnaA07g27400D and BnaC06g29530D in a variety of Xiaoyun (Geng et al., 2022; Wang et al., 2024), the phenotype of edited plant is petal attaching to the flower till pod mature and enhancing the force of silique dehiscence, which facilitated to preventing sclerotinia infection and improving mechanical harvesting. In the field of rapeseed breeding, high yields and other excellent traits are prerequisites. Semi-winter varieties, exemplified by varieties such as ZS11, K407, and Gangan are commercial varieties particularly widely cultivated or hybrid parents (Sun et al., 2017). However, plant regeneration in tissue culture are genotype dependent. It is possible to introduce the mutant alleles into core varieties or commercial cultivars by backcrossing integrating allele specific marker assistant selection.

In this study, we utilized the CRISPR/Cas9 system to precisely edit the two homologous IDA genes in *Brassica napus* (ZS11). Subsequently, we performed RNA-Seq analysis on the resulting *Bnaida* mutants and developed rapid identification primers for gene typing. Afterwards, we aimed to swiftly integrate the mutant loci into our breeding materials, including lines such as SW1-6, to develop a feasible implementation plan for gene editing combined with hybrid backcrossing breeding technology.

## 2 Materials and methods

### 2.1 Materials

The rapeseed cultivar ZS11 from our germplasm collection served as the transformation host. Tissue cultures were grown at 24 ± 2°C under 4500 lux illumination with a 16/8 h light/dark cycle. Both transgenic and Wild-Type (WT) plants were subsequently cultivated in greenhouse conditions at 24 ± 2°C with 6000 lux light intensity and identical photoperiods.

### 2.2 Vector construction and plant tissue culture

To design targets for *BnaIDA-A07/C06*, we used the CRISPR-P tool (http://cbi.hzau.edu.cn/CRISPR2/) and the RNA Folding tool (https://www.unafold.org/mfold/applications/rna-folding-form-v2.php). These tools helped us identify suitable target sequences. For genome editing in ZS11 rapeseed plants, we employed Agrobacterium tumefaciens (strain GV3101) to mediate transformation, following the protocol described by Rui Geng (Geng et al., 2022).

### 2.3 RNA extraction, cDNA synthesis, and qRT-PCR analysis

Total RNA was isolated from leaves and floral tissues by using Trizol reagent. cDNA was then synthesized using the HiScript III 1st Strand cDNA Synthesis Kit (R312-02, Nanjing Vazyme Biotech, China). Quantitative real-time PCR (qRT-PCR) was performed using the SYBR qPCR Master Mix (Q331-02, Nanjing Vazyme Biotech, China) on an Applied Biosystems^®^ QuantStudio^®^ 3 Real-Time PCR System (Thermo Fisher Scientific, USA). *BnaACTIN* was used as the reference gene. Relative expression levels were determined using the 2-ΔΔCt method, with calculations performed by the qRT-PCR instrument software.

### 2.4 DNA extraction and identification

Genomic DNA was extracted from the leaves and petals of both mutant and WT plants using the cetyltrimethylammonium bromide (CTAB) method. PCR amplification was performed using vector-specific primers, including U6, Cas9, and Hyg, followed by Sanger sequencing and Hi-TOM sequencing to obtain precise editing profiles (Sun et al., 2024). Specific identification primers were subsequently designed to amplify genomic regions encompassing the target sites via PCR.

### 2.5 Bioinformatic analysis

All RNA extraction and data processing procedures were conducted using BGI’s online website at https://report.bgi.com/ps/mrna/index.html. The raw sequence data reported in this paper have been deposited in the Genome Sequence Archive (Chen et al., 2021) in National Genomics Data Center (Members and Partners, 2024), China National Center for Bioinformation/Beijing Institute of Genomics, Chinese Academy of Sciences (GSA: CRA016493) that are publicly accessible at https://ngdc.cncb.ac.cn/gsa.

### 2.6 Scanning electron microscopy

Abscission zone (AZ) samples were collected from five distinct flowering positions of both WT and mutant plants. The tissues were fixed in 2.5% glutaraldehyde (w/v) dissolved in 0.2 M phosphate buffer (pH 7.2) at 4°C. After fixation, the samples were dehydrated using an ES-2030 freeze dryer (Hitachi) and affixed to carbon tape. A gold-palladium coating was applied using an E-1010 sputter coater (Hitachi). Imaging was performed with an S-3000N field emission scanning electron microscope (Hitachi) at an acceleration voltage of 5 kV.

## 3 Results

### 3.1 Acquisition of BnaIDA-ZS11 mutant plants

**Figure 1:**
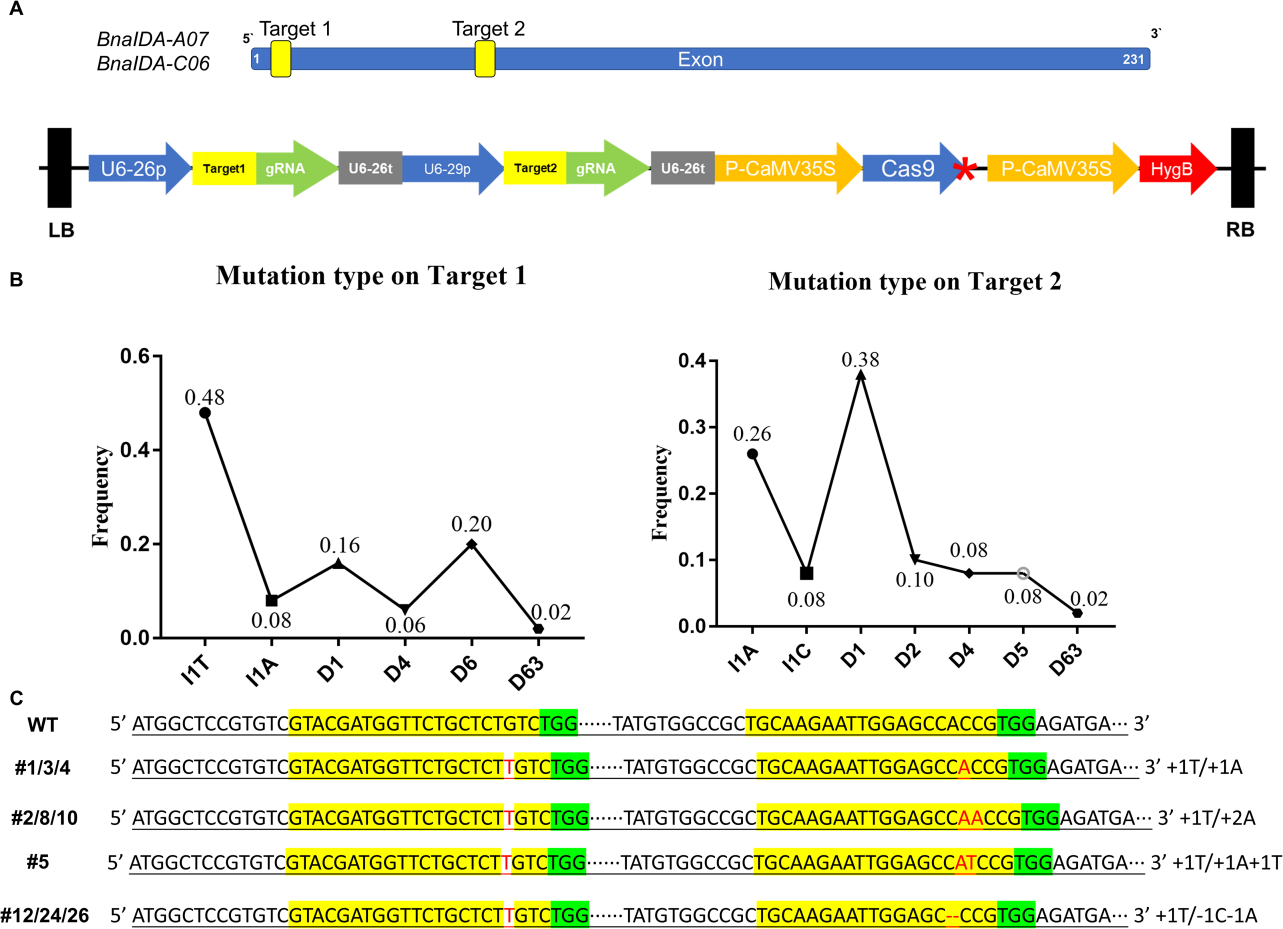
Gene editing strategy and analysis of BnaIDA-A07/C06-ZS11. **(A)** Schematic illustration of the CRISPR-Cas9 vector targeting the conserved coding sequence region of BnaIDA. **(B)** Mutation types and frequency at the sgRNA target sites in T0 mutants. The X-axis: I# and D# were the numbers of base pairs inserted and deleted at the sgRNA target sites. **(C)** showing mutation sites at two homologous copies of *T1-BnaIDA* (*BnaIDA-ZS11-A07/C06*). Targets were colored yellow. Red represented the edited base. PAMs were colored green. “-” indicated nucleotide deletions in the target sequence.

The gene and protein sequences of IDA were retrieved and analyzed using the *B. napus* database (https://yanglab.hzau.edu.cn/BnIR). To achieve simultaneous knockout of the *BnaIDA-A07*/*C06* genes, two target sites were strategically selected within the 5’ region of the coding sequence (CDS) to ensure efficient editing. These targets were designed to induce frameshift mutations, thereby causing premature termination of protein translation. Two guide RNA (gRNA) sequences targeting conserved regions were synthesized and incorporated into a CRISPR/Cas9 multiplex editing system (**Figure 1A**) (Zhang et al., 2019).

The pHSE401-BnaIDA plasmid was introduced into the hypocotyl of *B. napus* cultivar ZS11 via AF and tissue culture. Transformed callus were rapidly selected using hygromycin resistance, and 12 positive transgenic plantlets were obtained through tissue culture. Sanger sequencing of the T0 generation revealed a 48% frequency of a single T base insertion at target site 1, while at target site 2, a 38% frequency of single base deletion and a 26% frequency of single A base insertion were observed (**Figure 1B**). To identify the *BnaIDA-ZS11* mutant plants, we employed Sanger sequencing and Hi-TOM sequencing to analyze the editing outcomes in the T1 generation. These methods revealed a variety of editing events, including single or multiple base insertions and deletions (**Figure 1C**). In addition, we also 1000-seed weight, seed number per silique, plant height and branch number but found no significant difference between *Bnaida* and WT. This also indicates that our mutation did not bring any adverse agronomic traits (**Supplementary Figure S5**). Collectively, these results demonstrate the successful application of the CRISPR/Cas9 system to simultaneously knockout *BnaIDA-A07* and *BnaIDA-C06* in the ZS11 genome, with precise editing near the protospacer adjacent motif (PAM) sequence. These findings suggest that multiplex gene editing in *B. napus* is feasible and that the exogenous T-DNA could be segregated from the progeny through selfing, highlighting the potential of CRISPR/Cas9 for rapid crop breeding.

### 3.2 The *Bnaida* exhibited prolonged flowering period by affecting the isolation of AZ cells

**Figure 2:**
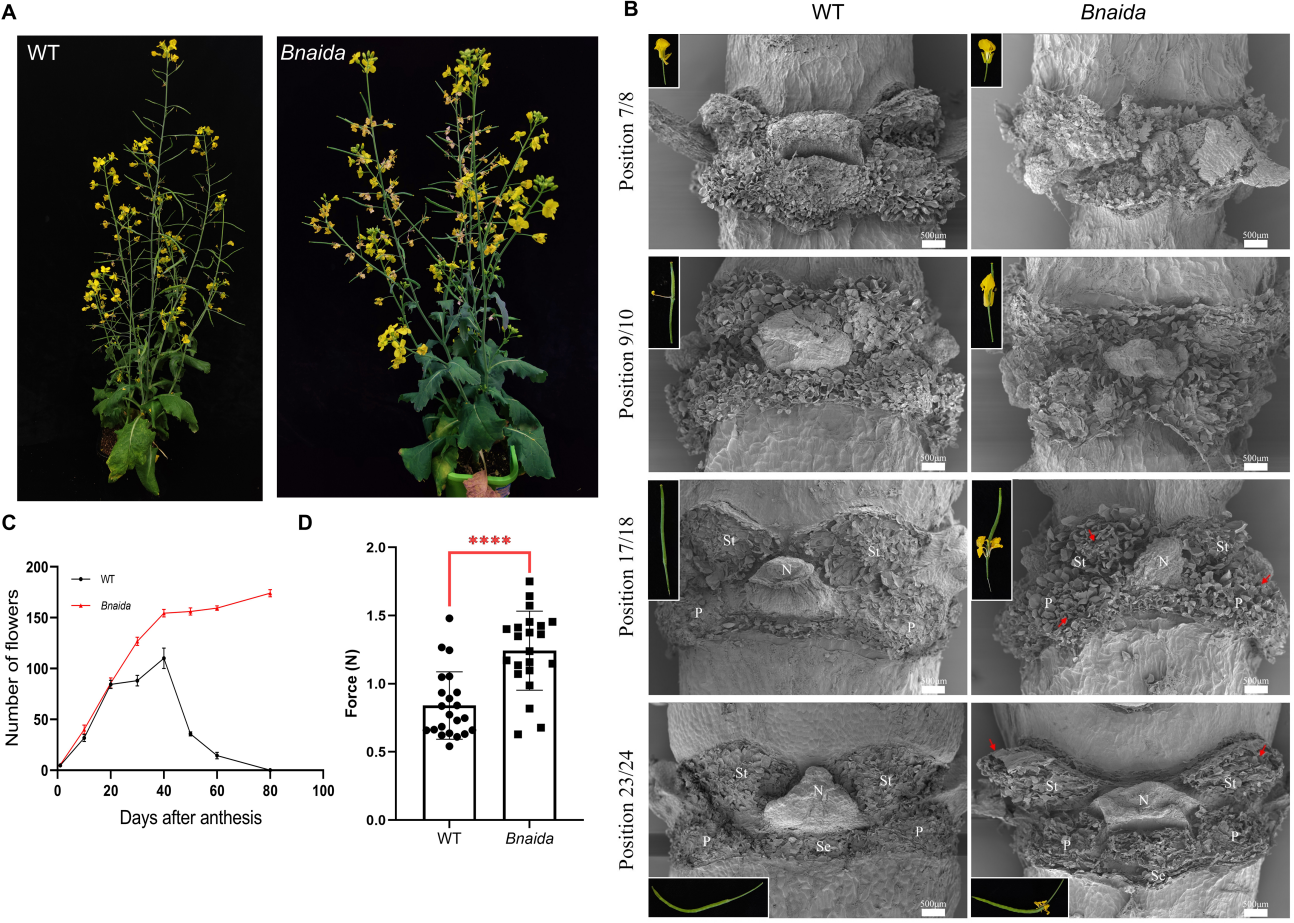
The *Bnaida* exhibited a prolonged flowering period by affecting the isolation of AZ cells. **(A)** *Bnaida* mutants are deficient in floral organ abscission. **(B)** SEMs of floral abscission zones in *Bnaida* and WT plants. Images were captured at the mid-region (position 1/2) of the primary inflorescence. St, Stamen AZ. Se, sepal AZ. N, Nectaries AZ. P, Petal AZ. The red arrows highlight the residual floral organs. Bars = 500 μm. **(C)** Quantitative statistics of WT and mutant flowers over 80 days. (n = 10, bars = SD). **(D)** Schematic diagram of texture analyzer and the force measurements of siliques opening. (n = 22, bars = SD; **** Student′s t-test, P < 0.0001).

The floral organs of the *Bnaida-ZS11* mutant remained attached from flowering through maturity, even as pods desiccated and petals age and lose moisture (**Figures 2A, C**). The IDA-HAE/HSL2 signaling pathway regulates organ separation by modulating abscission zone (AZ) cells activity. Silique dehiscence, a related process, involves AZ cell expansion (Stenvik et al., 2006). Notably, *BnaIDA-A07/C06* mutations significantly downregulated *CEL1*, an endoglucanase gene critical for cell wall degradation, potentially explaining the abnormal AZ cell separation in *Bnaida* mutants (**Figure 2B**).

Scanning electron microscopy of AZ cells at different developmental stages revealed that while WT and *Bnaida* floral organs remained attached before position 7/8, only *Bnaida* organs persisted beyond position 9/10 (Patterson and Bleecker, 2004). Post-abscission, WT AZ cells restored a flat surface, whereas *Bnaida* silique junctions retained residual stamen, petal, and sepal cells (**Figure 2B**). The *Bnaida* mutant exhibited higher tensile strength (1.75N) compared to WT (1.48N), suggesting enhanced silique integrity (**Figure 2D**). These findings demonstrate that *Bnaida-ZS11* prevents floral organ shedding and pod shattering by disrupting AZ cell transdifferentiation, highlighting its potential for breeding applications.

### 3.3 Comparative RNA-Seq analysis between *Bnaida* and WT lines

#### 3.3.1 Overview of the transcriptome of two lines

**Figure 3:**
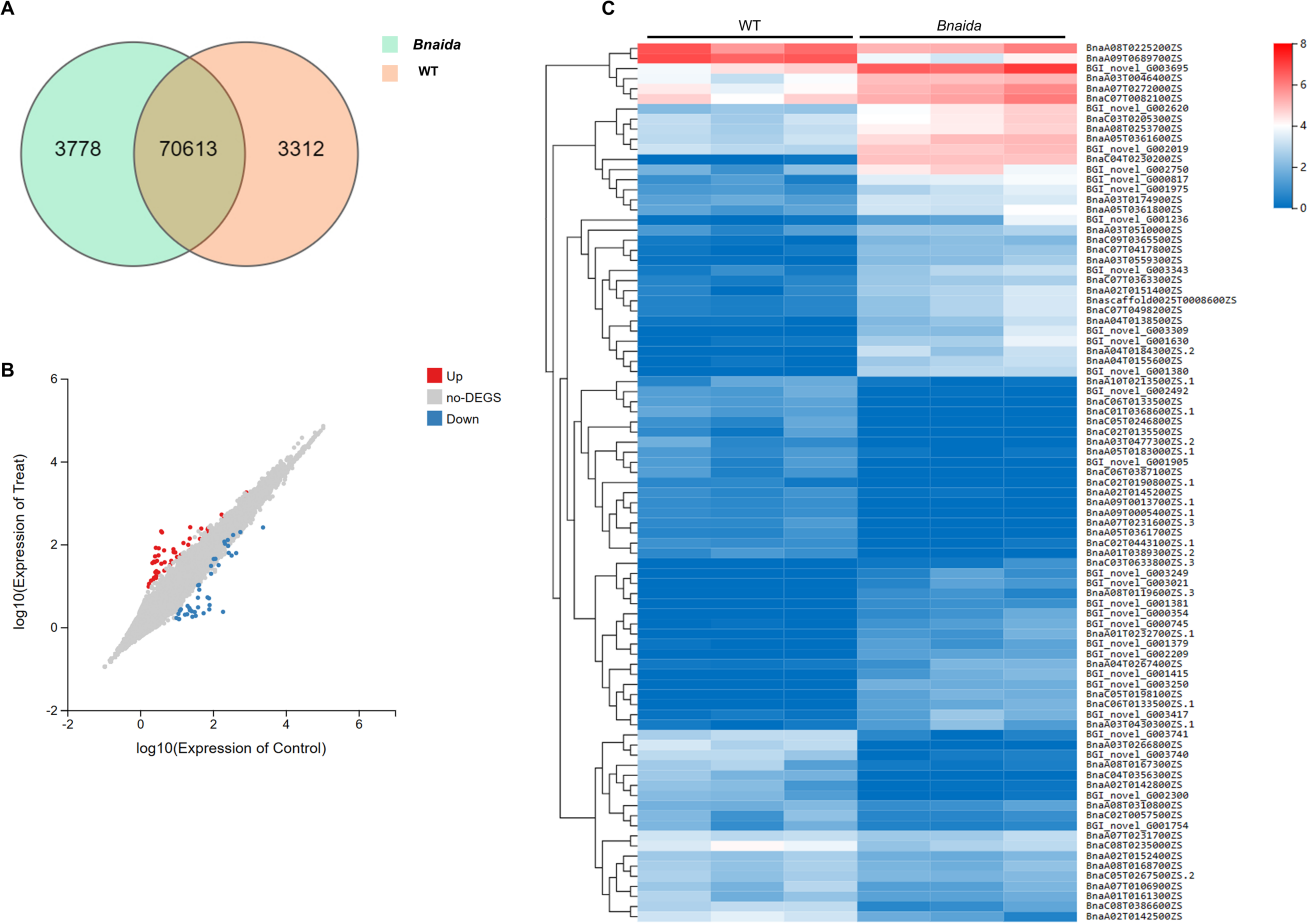
RNA-Seq and differential gene expression analysis in mutants and WT. **(A)** Venn diagram of mutant and WT. **(B)** The scatter plot. **(C)** Cluster heat map of differential gene expression. The redder the color, the higher the expression, and the bluer the color, the lower the expression.

For the further clarification of IDA-HAE/HSL2 signaling pathway and its downstream regulations, large-scale transcriptomic profiling was conducted using RNA-Seq in order to accurately assess differences in mRNA expression between mutants and WT. After filtrating out the raw reads with low-quality, the proportion of clean reads in raw reads ranged from 86% to 92% across all samples, with Q20 and Q30 scores exceeding 95% and 89%, respectively. The high base-calling accuracy confirmed the reliability and stability of the sequencing data. A total of 77,703 genes were identified as expressed above the threshold in the comparison between WT and *Bnaida* mutants. Among these, 3,778 genes exhibited exclusive expression in *Bnaida*, whereas 3,312 genes were uniquely expressed in the WT (**Figure 3A**).

Differentially expressed genes (DEGs) were identified using a threshold of false discovery rate (FDR) < 0.05 and |log2 fold change (FC)| > 1. Comparative analysis between the two groups revealed 91 DEGs, comprising total of 49 genes were up-regulated, while 42 genes were down-regulated (**Figures 3B, C**). To confirm the reliability of the identified DEGs, qRT-PCR was conducted using specific primers targeting 13 randomly selected DEGs and three top candidate genes (**Supplementary Table S1**). The results indicated that DEGs displayed expression patterns consistent with the RNA-seq data (**Supplementary Figure S1**).

#### 3.3.2 GO classification and KEGG enrichment analysis

**Figure 4:**
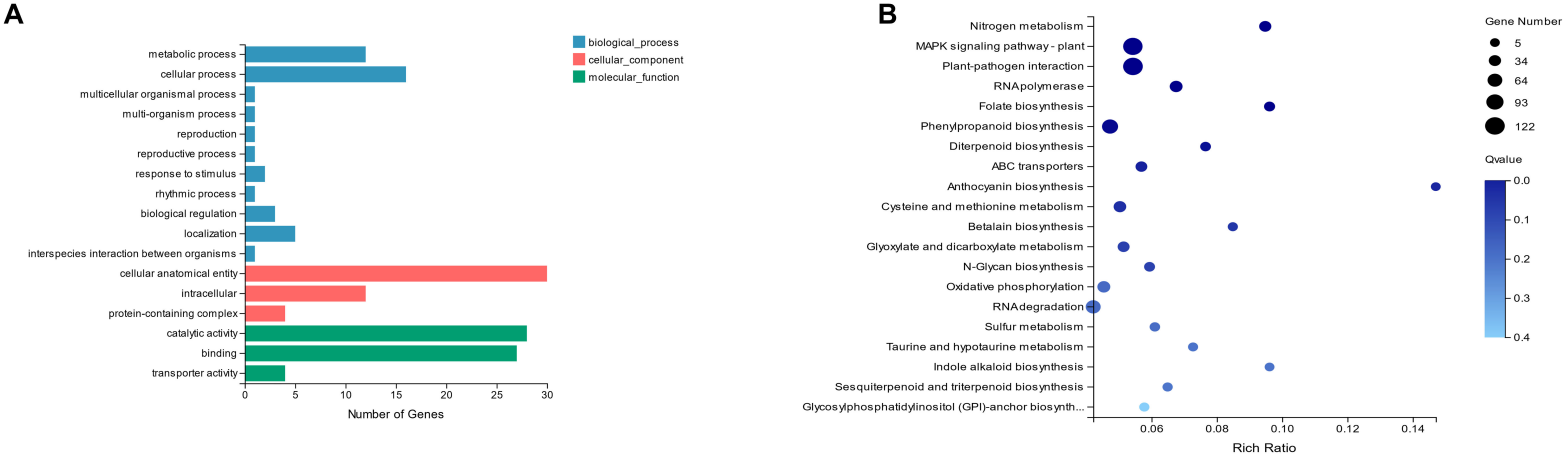
GO **(A)** and KEGG **(B)** Analysis of DEGs. The vertical axis indicates GO categories and KEGG pathways, while the horizontal axis shows the number of genes and enrichment ratios.

GO enrichment could classify differentially expressed genes into cellular components (CC), molecular functions (MF), and biological processes (BP) based on their functions. Among them, genes related to cellular process, cellular anatomical entity, and catalytic activity were significantly enriched (**Figure 4A**). The above functions are all involved in biosynthesis, cell membrane composition, and metabolism pathways in plants.

Kyoto Encyclopedia of Genes and Genomes (KEGG) pathway enrichment analysis revealed that the genes were predominantly enriched in the plant MAPK signaling pathway, phenylpropanoid biosynthesis, and plant-pathogen interaction pathways. (**Figure 4B**). To further elucidate the expression patterns of the IDA signaling pathway in *B. napus* and validate the reliability of the RNA-Seq data, we analyzed the expression levels of associated DEGs**. (Supplementary Figure S2**). In addition, the mutation of *BnaIDA-A07/C06* resulted in the significant down-regulation of the expression of *CEL1*, an endoglucanase gene associated with cell wall degradation, which probably explains the abnormal separation of cells in the AZ of *Bnaida*.

### 3.4 Rapid identification of *Bnaida* mutants and gene editing combined with breeding technology

**Figure 5:**
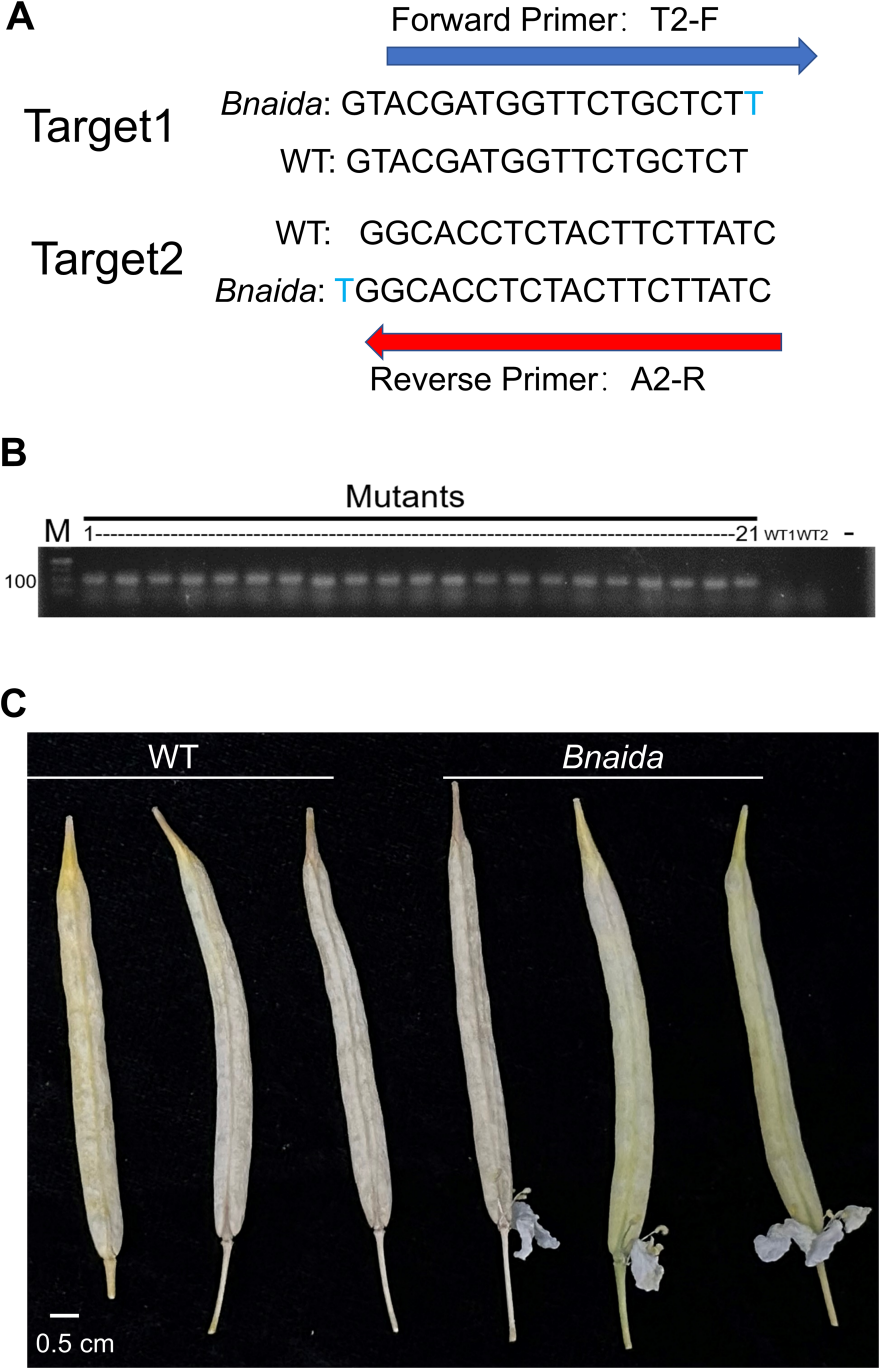
Rapid identification of *Bnaida* mutants. **(A)** Design of primers for rapid identification of *Bnaida* mutants. **(B)** Rapid identification of *Bnaida* mutants. M: DL500 Marker(bp); -: Negative control (ddH_2_O); WT-1/2: Wild Type (The leaf genome of the ZS11). **(C)** *Bnaida* fruits retain floral organs indefinitely.

Insertion of a single nucleotide T in the target site of BnaIDA-A07/C06 could lead to frameshift mutations, resulting in early termination of translation and eventually function loss of the BnaIDA peptide. (**Supplementary Figure S3**) To easily distinguish WT and *Bnaida* mutant alleles and quickly identify mutants with editing at two targets, allele-specific primers were developed (Wu et al., 2020). As depicted in **Figures 5A, B**, specific primers have been designed to efficiently distinguish between dominant and recessive alleles. Homozygous genome editing was verified using primer pairs T2-F/A2-R, and the corresponding phenotype displayed non-petal abscission. (**Figure 5C**). Therefore, WT and homozygous mutant plants could be readily differentiated by applying primer genotype analysis.

**Figure 6:**
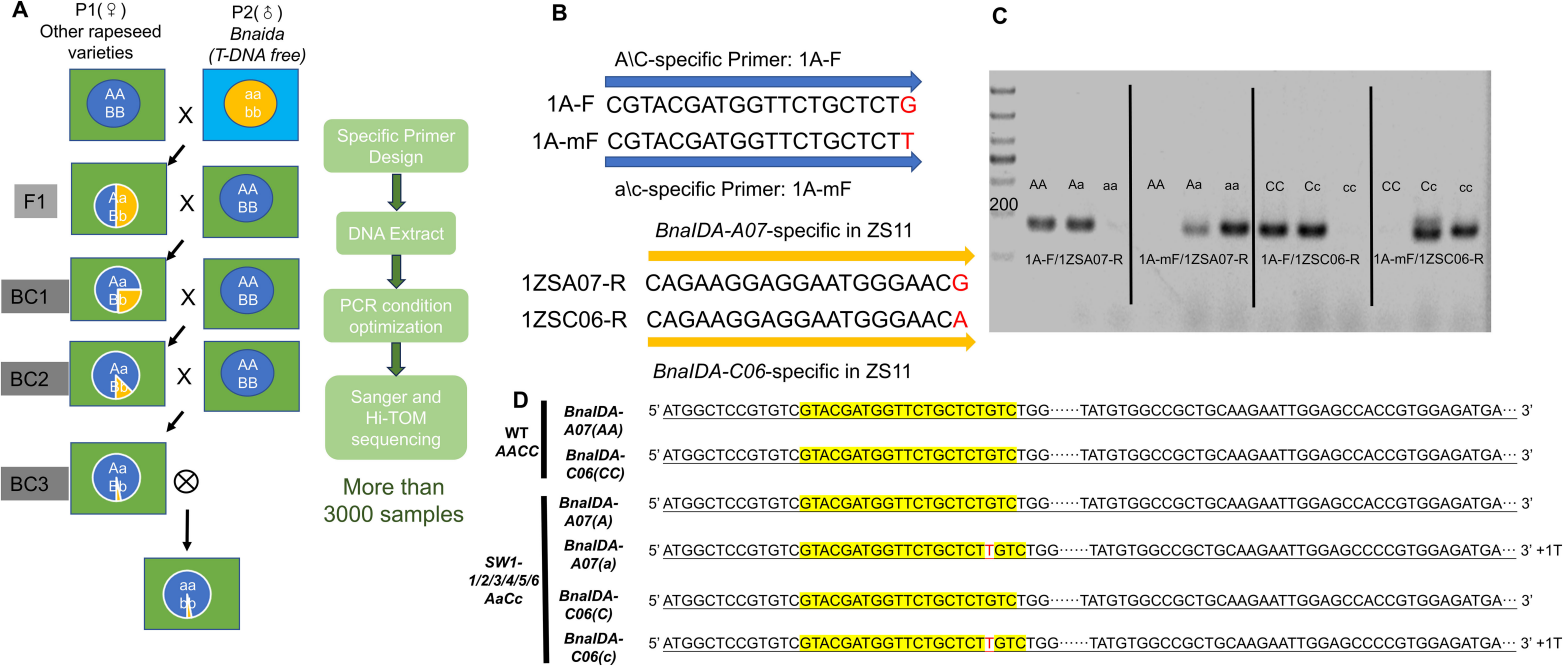
Gene editing combined with breeding technology. **(A)** Strategy diagram for cross and backcrossing breeding of *Bnaida* (T-DNA free) with other rapeseed varieties. **(B, C)** Primer design diagram for distinguishing dominant and recessive alleles A-A/C-C, as well as distinguishing *BnaIDA-A07* and *BnaIDA-C06* genes. **(D)** Sanger sequencing and Hi-TOM of BC_2_ generation plants with AaBb genotype. Targets were colored yellow. Red represents the inserted base.

In the process of rapeseed breeding, the initial steps are to hybridize and backcross mutants with diverse varieties to generate successive generations of offspring, such as F_1_, BC_1_, BC_2_, BC_3_.etc. Following this, successful integration of the target mutation site into the genome of the chassis variety is achieved through self-pollination segregation (**Figure 6A**). To propel the breeding process forward, precise identification of heterozygous individuals with the AaBb genotype becomes extremely important, serving as a pivotal step to ensure the seamless advancement of subsequent backcrossing experiments.

To precisely discern between the dominant and recessive alleles A-a/C-c, we implemented a specialized design at the 3’ end of the forward primer, termed 1A-F and 1A-mF, respectively. Similarly, for the reverse primer, distinctive designs were employed at the 3’ end to differentiate between *BnaIDA-A07* and *BnaIDA-C06*, termed 1ZSA07-R and 1ZSC06-R, respectively (**Figure 6B**). We successfully hybridized and cross-bred *Bnaida* (T-DNA free) We successfully crossed Bnaida (T-DNA-free) plants with SW1 to SW6 chassis varieties, generating 70 F1 progeny. These offspring were subjected to PCR, Sanger sequencing, and Hi-TOM analysis, leading to the production of 860 BC1 plants through backcrossing of screened positive individuals. Following repeated identification, approximately 150 target plants were selected for further backcrossing with diverse parental lines. Using heterozygous allele-specific PCR primers, we accurately and efficiently identified individuals with the AaBb genotype based on the analysis of over 3,000 samples (**Figure 6C**, 6D). This new strategy and germplasm not only provide reliable heterozygous individuals for subsequent experiments but also present a more economically feasible approach for breeding work.

## 4 Discussion

### 4.1 BnaIDA-driven regulation of organ abscission and cell wall degradation in rapeseed

Plant abscission is regulated by various signaling pathways, with phytohormones playing a central role. In *Arabidopsis*, exogenous application of NAA accelerates fruit abscission, while abscisic acid (ABA) modulates the process indirectly by influencing ethylene biosynthesis (Butenko et al., 2006; Niederhuth et al., 2013). Mutations in genes associated with ethylene perception or auxin signaling often result in delayed abscission (Ellis et al., 2005). Furthermore, abscission is intricately linked to senescence, a developmentally programmed process governed by a multi-layered regulatory network. This process is further amplified by interconnected feedback loops involving CDF4, ABA, and reactive oxygen species (ROS), which collectively promote leaf senescence and floral organ shedding (Xu et al., 2020). Additionally, IDA-HAE/HSL2 signaling pathway plays a critical role in floral abscission. IDA peptide serves as a ligand recognized by HAE/HSL2 receptors with various coreceptors such as SOMATIC EMBRYOGENESIS RECEPTOR KINASES (SERKs), in forming a plasma membrane-localized complex. On account of the phosphorylation is initiated by ligand receptor binding, the MITOGEN-ACTIVATED PROTEIN KINASE (MAPK) cascade is activated. This cascade subsequently regulates transcription factors such as *BREVIPEDICELLUS (BP)/KNAT* and *KNAT2/6*, ultimately modulating cell wall hydrolase activity and controlling floral organ abscission (Shi et al., 2011; Meng et al., 2016). CRISPR/Cas9-mediated knockout of *BnaIDA-A07/C06* in *B. napus* confers multifaceted agronomic benefits, including enhanced silique shattering resistance and prolonged floral retention for agrotourism appeal (Geng et al., 2022). These two homologous genes need to be knocked out simultaneously to obtain the phenotype (Wu et al., 2022), so the mutant screening in this study is the necessary process in the identification of the mutant. It is necessary to obtain heterozygotes of AaBb, and in future research, it is possible to obtain recessive homozygous mutants of aabb through self-crossing in order to obtain the desired phenotype. The integration of CRISPR edited *Bnaida* loci into excellent rapeseed lines such as SW1-6 reflects a shift in breeding ideas and efficiency. *Bnaida* can be combined with resistance alleles such as *BnaC07. GLIP1* or high oil traits *BnaTT2/8* to achieve aggregation of excellent traits in a single variety through multiple editing (Ding et al., 2024; Li et al., 2024). The allele specific genotyping system developed here has been validated in six commercial varieties, achieving a 95% accuracy in identifying heterozygous AaBb genotypes. The CRISPR strategy efficiently targets functional homologs in polyploid crops without detectable off-target effects, ensuring stable agronomic performance. This multifunctional genome-editing approach addresses yield security and economic value, positioning *Bnaida* mutants as a sustainable germplasm resource for rapeseed improvement.

### 4.2 Advances in plant regeneration and transformation

The AP2/ERF transcription factor WIND1 is crucial for plant tissue regeneration, enabling *de novo* root regeneration without auxin pretreatment. In *Brassica napus*, *AtWIND1* activation enhances shoot regeneration, while its interaction with *LEAFY COTYLEDON2* (LEC2) promotes embryogenic callus formation, highlighting its role in tissue-specific regeneration (Iwase et al., 2015). Recent advances in maize transformation utilize the *PLTP* and *Axig1* promoters to drive *Bbm* and *Wus2*, respectively, enabling genotype-independent somatic embryogenesis without callus formation (Lowe et al., 2016). This system achieves transformation efficiencies of 9%–224% and produces normal, fertile plants across maize inbred lines. In the recalcitrant maize line PHH5G, *Bbm* and *Wus2* overexpression achieved transgenic callus formation in >40% of explants, with most differentiating into healthy plants. This method also succeeded in mature seed embryos and leaf explants, demonstrating its potential to overcome genotype-dependent transformation barriers (Lowe et al., 2018). Although advances in plant regeneration and transformation have been achieved, their application remains constrained by significant variations in transformation efficiency among maize inbred lines, incomplete understanding of *Bbm/Wus2* molecular regulatory mechanisms and long-term stability, and the failure of over half of *PHH5G* explants to respond. Current methods rely on specific promoter combinations and focus on explants at particular developmental stages, while their cross-species applicability and adaptability to diverse tissues still require validation.

### 4.3 The Role of BnaIDA in cell wall degradation and abscission zone function

Organ abscission is crucial in plants, which could speed up the metabolism of plants or abandon damaged tissues and organs in time. The abscission of various tissues and organs is due to the function of abscission zone cells. Among them, floral organs and pods are the organs that will be separated in the normal life activities of rapeseed and will be affected by the cells in the abscission zone. After flowering and pollination, the petals and stamens of Arabidopsis gradually apoptosis, and the pistil develops into a pod. Then the silique elongated continuously, and the replum in the separation layer began to crack; the inner pericarp layer was lignified and the outer pericarp layer decomposed. Finally, the pericarp continues to turn yellow, water is lost, and the seed coat turns yellow-brown, entering the post ripening stage (Yu et al., 2020). This series of programmed events will lead to cell wall degradation, autolysis of non-lignified layer and mechanical tension between lignified cells in horn peel and non-lignified parenchyma cells in the pseudoseptum. Finally, the valve is separated from the embryo seat frame, resulting in seed release (Meakin and Roberts, 1990). *B.napus* and *Arabidopsis* belong to Cruciferae, and the organ abscission process of rapeseed is also controlled by the cells in the detachment area. Our study shows that *Bnaida* mutant does affect the separation of cells in the detachment area (**Figure 2**). At the same time, we observed a significant downregulation in the expression of *CEL1*, an endoglucanase gene involved in cell wall degradation (**Supplementary Figure S2**). The results demonstrated that the loss of BnaIDA-A07/C06 function in *B. napus* resulted in the downregulation of downstream cell wall-degrading enzymes, thereby disrupting the temporal and spatial regulation of AZ dehiscence.

### 4.4 Challenges and prospects in rapeseed breeding and genome editing

Rapeseed breeding necessitates a wide array of agronomic traits, many of which are governed by multiple genes (Du, 2025). Given the diverse ideal traits and genotypes exhibited by different varieties, enhancing yield potential requires aggregating these excellent traits through gene combination. Typically, this entails amalgamating the desirable traits of multiple individuals via hybridization and backcrossing. However, traditional breeding methods primarily rely on phenotype selection, resulting in prolonged breeding cycles alongside low efficiency and accuracy (Gu et al., 2024). When designing primers for heterozygous and homozygous rapeseed, it is crucial to not only differentiate editing sites but also distinguish the genotype. For instance, in the case of *BnaIDA - A07/C06*, besides confirming mutation site insertion, we should identify different SNPs on the *BnaIDA-A07* and *Bna-C06* genes for primer design. Moderately raising the primer binding annealing temperature can lower false-positive results, yet excessive increase may hinder amplification. A key innovation lies in the development of allele-specific primers for rapid genotyping, enabling efficient identification of heterozygous/homozygous individuals during backcrossing with commercial lines like SW1-6 (Roscher-Ehrig et al., 2024). This approach reduced breeding cycles by more than 30% compared to traditional phenotypic selection, as demonstrated by the successful introgression of *Bnaida* loci into 860 BC1 plants.

Currently, genome editing technology based on CRISPR technology is a powerful tool for crop improvement, capable of creating new variations in functional genes, which is in stark contrast to the traditional breeding method that relies mainly on natural variation (Zhai et al., 2019; Hu et al., 2024). Nonetheless, technical constraints persist. While Agrobacterium-mediated transformation remains the most widely employed transgenic method in rapeseed, its efficiency in genome editing falls short of practical application demands, which is relatively not advantageous a handful of easily transformable varieties (Gao, 2021; Gu et al., 2024). Many varieties integral to breeding could not undergo effective transformation and tissue culture. This method has achieved a nearly hundredfold reduction compared to Sanger sequencing. Despite multiple optimizations and validation through thousands of experiments, with an accuracy rate exceeding 95%, some false positives persist (**Supplementary Figure S4**). These discrepancies may arise from variations in PCR instruments, aerosol contamination and temperature fluctuations. In future research, after obtaining more stable hybrid progeny, we will adopt a combined strategy of foreground and background selection. This integrated approach will enable comprehensive genotyping of mutants and accelerate the production of homozygous offspring through enhanced selection efficiency.

## 5 Conclusion

In this study, we integrated traditional breeding methods with genome-editing techniques to enhance our breeding strategies. One key challenge we faced was the efficient identification of heterozygous individuals. To address this, we designed a series of rapid identification primers based on the strict pairing principle at the 3’ end of the primers. These primers were subsequently validated using Sanger and Hi-TOM sequencing techniques, enabling us to accurately determine whether the backcross lines contained the AaBb genotype. Additionally, we performed RNA-Seq analysis and scanning electron microscopy on *Bnaida* mutants. These analyses will provide deeper insights into the *Bnaida* mutants and facilitate their application in variety breeding.

## Data Availability

The datasets presented in this study can be found in online repositories. The names of the repository/repositories and accession number(s) can be found in the article/**Supplementary Material**.

## Supplementary material

Supplementary Figure 1 The relative expression level measured by RNA-Seq and qRT-PCR.

Supplementary Figure 2 qRT-PCR analysis of the IDA-HAE/HSL2 pathway relative genes expression. Quantitative RT-PCR expression analysis of *BnaHAE*, *BnaHSL2*, *BnaBP/KNAT1*, *BnaMAPK6*, *BnaKNAT2*, *BnaKNAT6* and *BnaCEL1* in the WT and *Bnaida*.

Supplementary Figure 3 Analysis of frameshift mutation.

Supplementary Figure 4 PCR identification results.

Supplementary Figure 5 Comparison of main agronomic traits between *Bnaida* and WT.

Supplementary Table 1 Primers.
