# Supplementary figures and images for: CRISPR mutant rapid identification in B. napus: RNA-Seq functional profiling and breeding technology application
Source: Front Plant Sci. 2025 Apr 22;16:1572020. doi: 10.3389/fpls.2025.1572020 (PMC12052763; doi:10.3389/fpls.2025.1572020)

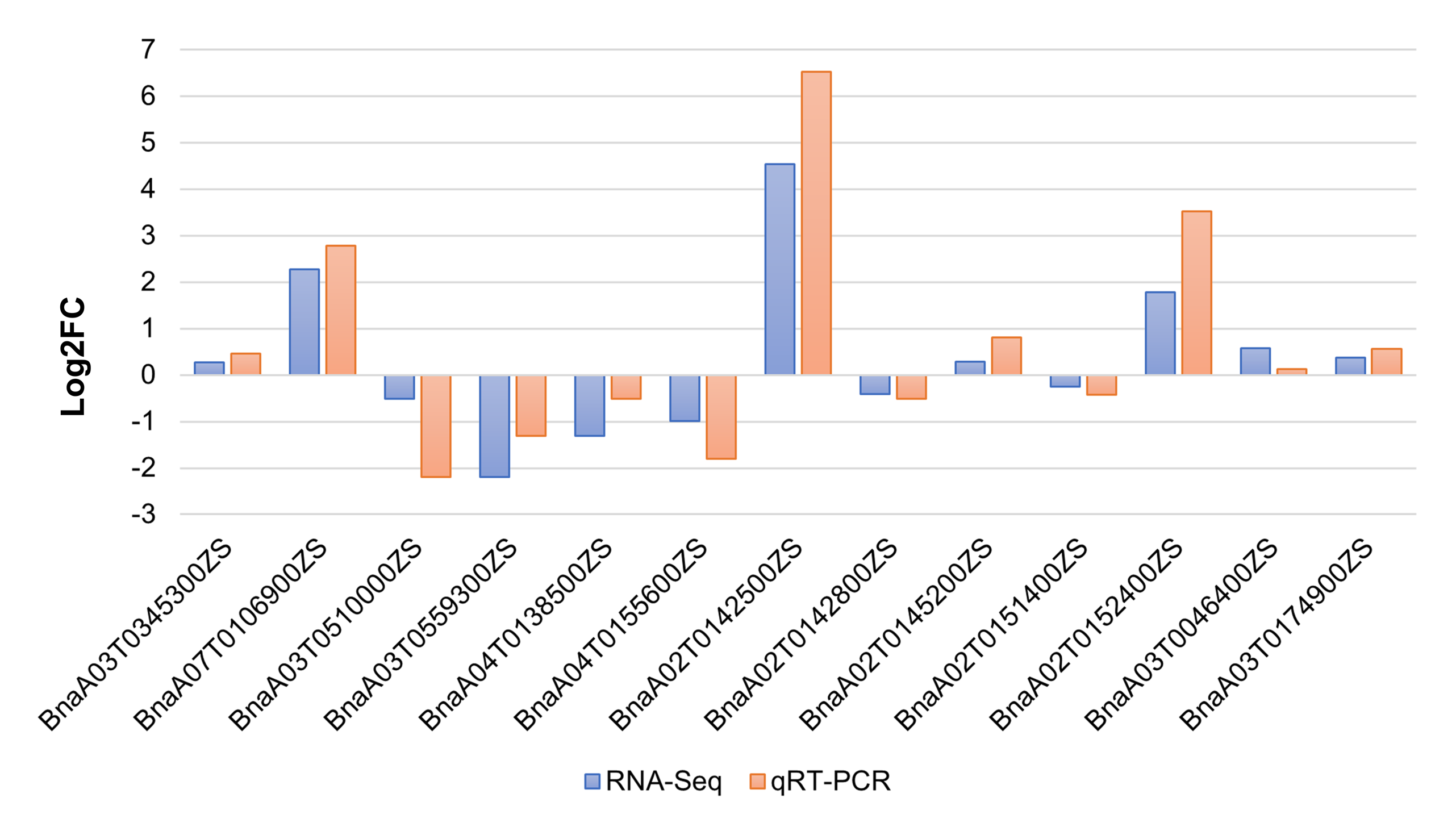

Supplement: Supplementary Figure 1 — The relative expression level measured by RNA-Seq and qRT-PCR. [file Image1.tif]

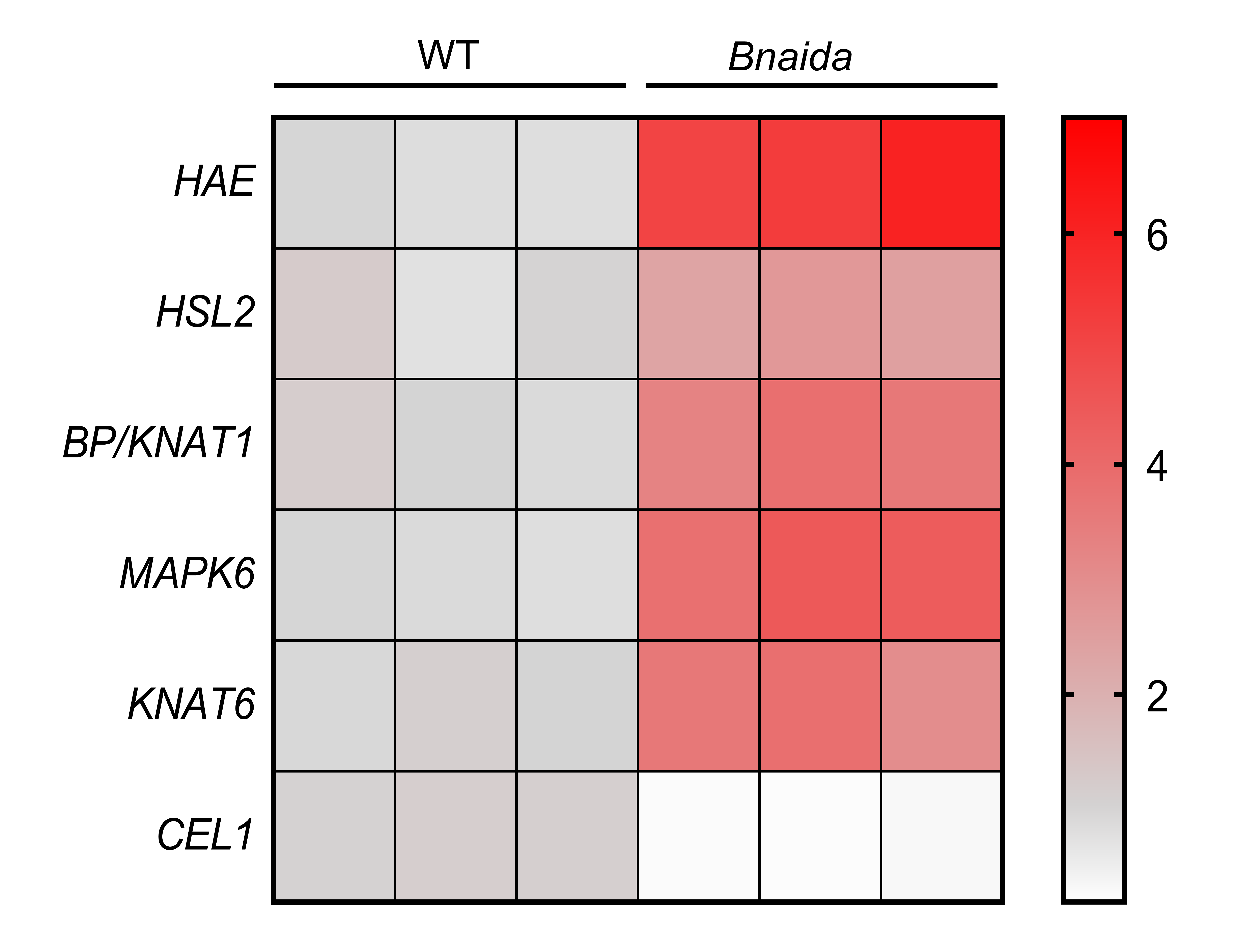

Supplement: Supplementary Figure 2 — qRT-PCR analysis of the IDA-HAE/HSL2 pathway relative genes expression. Quantitative RT-PCR expression analysis of BnaHAE, BnaHSL2, BnaBP/KNAT1, BnaMAPK6, BnaKNAT2, BnaKNAT6 and BnaCEL1 in the WT and Bnaida. [file Image2.tif]

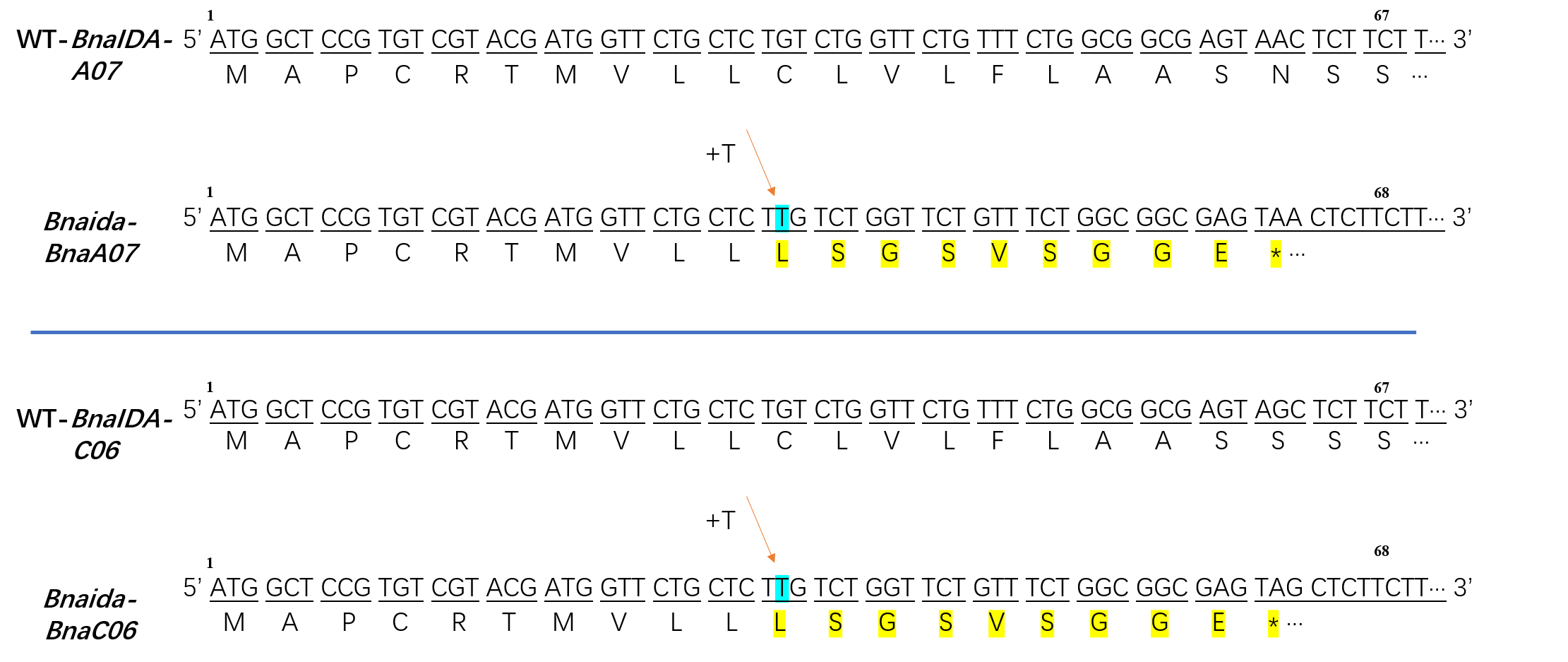

Supplement: Supplementary Figure 3 — Analysis of frameshift mutation. [file Image3.tif]

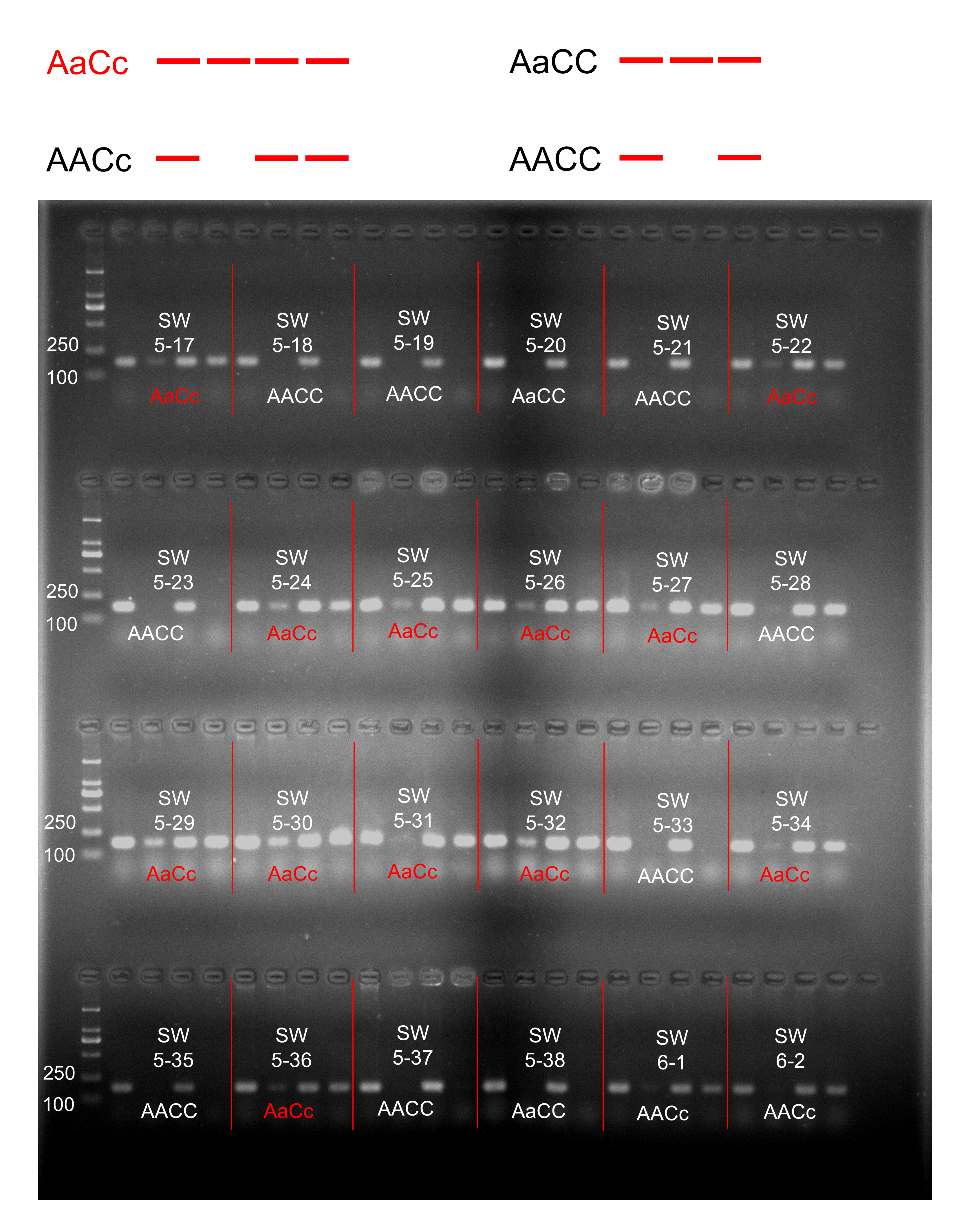

Supplement: Supplementary Figure 4 — PCR identification results. [file Image4.tif]

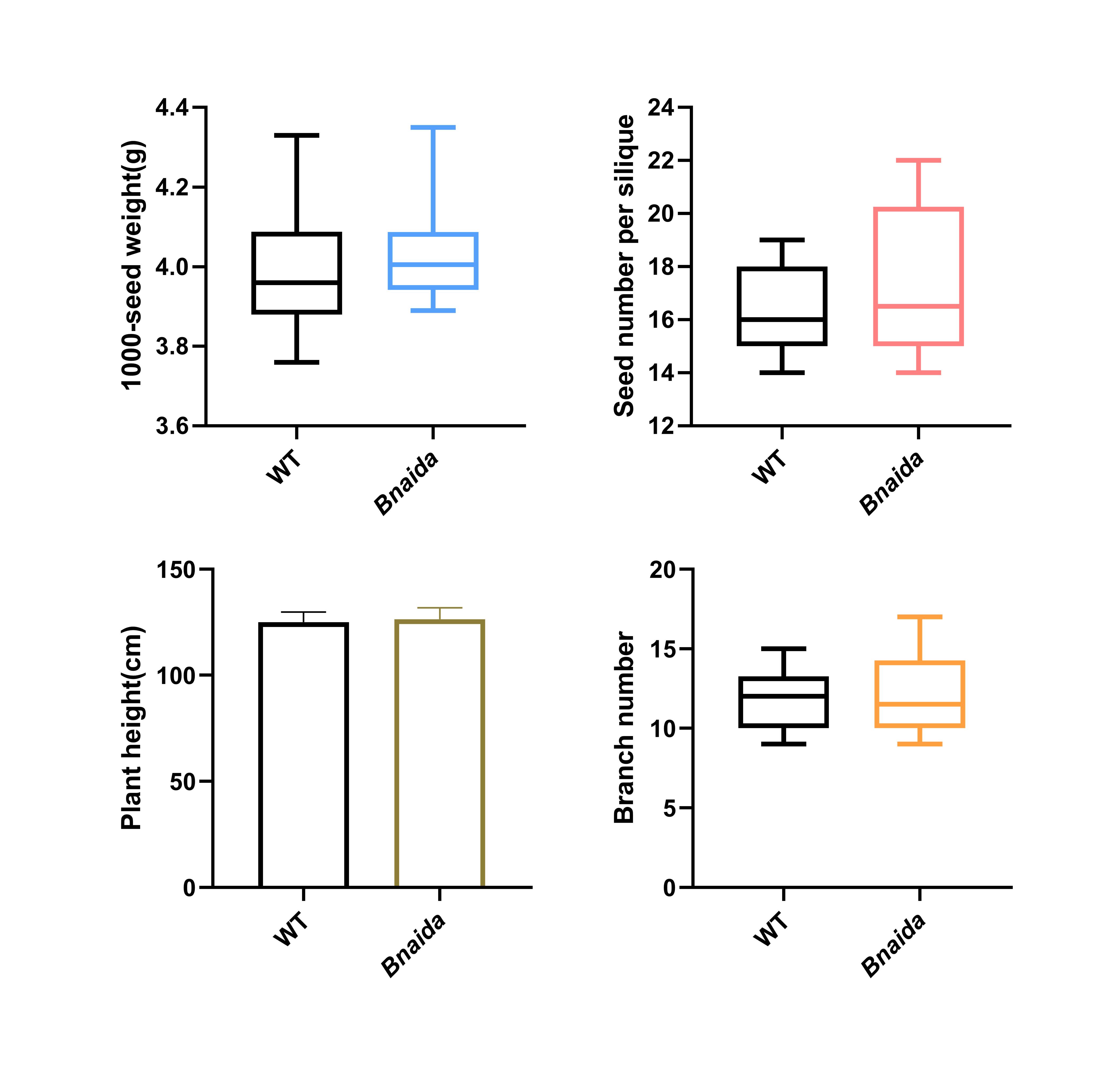

Supplement: Supplementary Figure 5 — Comparison of main agronomic traits between Bnaida and WT. [file Image5.tif]
